# Ethnopharmacokinetic- and Activity-Guided Isolation of a New Antidepressive Compound from Fructus Aurantii Found in the Traditional Chinese Medicine Chaihu-Shugan-San: A New Approach and Its Application

**DOI:** 10.1155/2012/607584

**Published:** 2012-02-01

**Authors:** Rong Fan, Xi Huang, Yang Wang, Xiao Chen, Ping Ren, Hui Ji, Ying Xie, Yingjin Zhang, Wei Huang, Xinjian Qiu, Zhaoqian Liu, Honghao Zhou, Lan Fan, Lichen Gao

**Affiliations:** ^1^Laboratory of Ethnopharmacology, Institute of Integrated Traditional Medicine and Western Medicine, Xiangya Hospital, Central South University, Changsha 410008, China; ^2^National Key Clinical Specialist Vocational School of TCM Encephalopathy, Xiangya Hospital, Central South University, Changsha 410008, China; ^3^TCM Pharmacogenetics Laboratory, Central South University, Changsha 410008, China; ^4^Jiangxi Qingfeng Pharmaceutical Company Ltd, Ganzhou 341000, China; ^5^Institute of Clinical Pharmacology, Central South University, Changsha 410008, China

## Abstract

*Aims*. We aimed to identify an antidepressive compound found in traditional Chinese medicine (TCM) by a new approach called ethnopharmacokinetic- and activity-guided isolation (EAGI). *Methods*. The new approach targets an unknown chromatographic peak produced by an absorbed compound found in oral Chaihu-Shugan-San (CSS) taken by patients with depression. Once the compound was isolated from Fructus Aurantii (FA), spectral data was employed to identify the compound. The effects of this compound, FA, and CSS on depressive behaviors were investigated. *Results*. The identified compound was merazin hydrate (MH) according to the new approach. MH, FA, and CSS significantly reduced immobility time and increased locomotor activity. The effects of MH, FA and CSS were similar to Fluoxetine at high doses. *Conclusion*. MH, a compound whose antidepressive effect is similar to FA and CSS, was isolated for the first time from FA via targeting its corresponding unknown chromatographic peak, and its antidepressive effect was compared with FA or CSS. These findings highlight the potential for drug R&D and pharmacological research of *∼*100,000 TCMs.

## 1. Introduction

Currently, drug R&D is highly challenging for scientific and economical reasons because of the high rate of failure, massive spending, and lengthy research period (1960s) [1]. Many phytochemicals have been isolated by activity-guided isolation (AGI) from herbs or plants [2] and shown to exhibit poor pharmacokinetic absorption [3]. There is limited data regarding the absorption properties of the compounds isolated from their parent herbs [2] as well as bioactive comparisons with the parent herbs [2].

It is important to determine a method that can successfully hit on a promising lead from a single herb or traditional Chinese medicine (TCM), which has been clinically used for more than 8000 years [4]. Interestingly, unknown chromatographic peaks in the blood and urine obtained from subjects who orally consumed TCMs provided the first leads [5]. During our pharmacokinetic study, an unknown chromatographic peak also appeared in blood samples 30 min after patients had been dosed with Chaihu-Shugan-San (CSS), a famous TCM which has been used for several centuries to improve some symptoms similar to depression [6]. Our goal was to isolate the pharmacokinetic compound corresponding to the unknown chromatographic peak and determine the parent herb. The ethnopharmacokinetic- and activity-guided isolation (EAGI) method allows for the identification of this compound.

We aimed to isolate a new antidepressive compound from a herb in CSS via targeting the unknown absorbed compound found in the blood from patients with depression following oral CSS and comparing its activity with the parent herb and CSS.

## 2. Experimental

### 2.1. Crude Drugs, Chemicals, and Reagents

CSS contains Bupleurum Root, Pericarpium Citri Reticulatae, Rhizoma Chuanxiong, Rhizoma Cyperi, Fructus Aurantii (FA), Paeonia, and Radix Glycyrrhizae in a ratio of 8 : 5 : 5 : 5 : 5 : 3 : 2-based dry weight. All of these compounds were purchased from the pharmacy at Xiangya Hospital (Changsha, China) and identified. The voucher specimens were deposited at the Laboratory of Ethnopharmacology in Xiangya Hospital. The 7 herbs were boiled twice in water (1 : 12, g/mL) for 1 h. The blended supernatants of two extracts were then lyophilized (the yield = 18.2%) for each of the herbs treated. FA, which had been known to contain an unknown peak, was lyophilized for pharmacological study, and its yield was 12.6%. The lyophilized powders of CSS and FA were stored at 4°C prior to use. The powders were dissolved in distilled water (CSS, 0.03 g/mL and FA, 0.002 g/mL (*w* : *v*)) prior to analysis pharmacological experiments.

 Albiflorin, ferulic acid, paeoniflorin, liquiritin, naringin, hesperidin, Neohesperidinn, isoliquiritigenin, glycyrrhizic acid, alpha-cyperone, and 18-bata-glycyrrhizic acid were purchased from the National Institute for the Control of Pharmaceutical and Biological Products (Beijing, China) and used as reference chemicals to exclude known compounds in CSS as part of the isolation procedure. Fluoxetine, which is an accepted positive antidepressive control agent, was purchased from Pharmacy of Xiangya hospital. Acetonitrile and methanol (HPLC grade) were obtained from the Tedia Company, Inc, Fairfield, Ohio (USA). The other reagents were of analytical grade. House triple-distilled water from silica glass equipment was also used. All of the samples for UPLC or HPLC were filtered through 0.22 *μ*m film prior to injection in the UPLC or HPLC.

### 2.2. Experimental Design

The EAGI approach included targeting the unknown chromatographic peak absorbed from the gut following oral consumption of CSS by depressive patients and ascertaining its herbal source. Then the unknown compound was isolated and identified and its antidepressive effects were compared with its parent herb or CSS.

### 2.3. Targeting an Unknown Chromatographic Peak in Patient Plasma and Detecting Its Herbal Source

#### 2.3.1. The Patient Enrollment and Administration

The study protocol was approved by The Medical Ethics Committee of Xiangya Hospital, China. Three patients experiencing a major depressive episode without psychotic features (males; mean age, 25.67 years; mean body weight, 62.2 kg) were enrolled in the present study according to a previously published protocol [7]. Informed consent was obtained from the patients. The oral dose of CSS (0.52 g/kg) in the present study is consistent with a previously published protocol [8]. The lyophilized powder of CSS was resolved to 1 g/mL (*w* : *v*) for each patient. The patients fasted for 12 h with free access to water prior to drug administration.

#### 2.3.2. Sample Collection and Preparation

Blood samples (5 mL) were obtained before and 30 min after oral CSS via venipuncture. The plasma (2 mL) and methanol (3.4 mL) were placed in a centrifuge tube (5 mL). The solution was mixed well by ultrasound vortexed for 5 min. Then the denatured protein precipitated and was separated by centrifugation at 3000 g for 15 min at 4°C. The supernatant was transferred to another tube and evaporated to dryness in a water bath at 50°C under a stream of nitrogen. The residues were reconstituted in 50 *μ*L of methanol with vortexing for 1 min, and the centrifugation procedure was repeated. Then 6.0 *μ*L of the methanol solution was injected into UPLC for analysis.

#### 2.3.3. UPLC-MS Determination of an Unknown Peak via Excluding the Known Compounds

UPLC-MS/MS contained a Waters Acquity UPLC system equipped with a quaternary solvent delivery system, an autosampler fitted with a 10 *μ*L loop and a PDA optical detector for ultraviolet wavelengths (190~500 nm). The separation was carried out on an acquity BEN C_18_ column (100 × 2.1 mm, 1.7 *μ*m, Waters, USA). The mobile phase consisted of a solution of acetonitrile (70–85%) and 0.5% acetic acid (30–15%) which was prepared daily and degassed prior to use. The flow rate of the pump was 0.5 mL/min, and the column temperature was set to 25°C. During the analyses, 6 *μ*L of sample was injected by the autosampler. The MS was performed on a Finnigan TSQ (San Jose, CA, USA) mass spectrometer equipped with an APCI interface. Mass spectrometric conditions were optimized to achieve maximum sensitivity. The APCI conditions were as follows: corona discharge voltage, 4.5 kV; heated capillary temperature, 330°C; nebulization temperature, 450°C; nitrogen was used both as sheath gas (70 psi, 1 psi = 6894.76 Pa) and auxiliary gas (25 a.u.). Argon was used as the collision gas with a collision energy of 35 V. The data was collected and processed using Empower TM Software.

Qualifications for known and unknown compounds from herbs of CSS were performed in multiple channels in the selected ion monitoring (SIM) mode using target ions at [M-H]^−^ m/z 479 for albiflorin [9], [M-H]^−^ m/z 449 for paeoniflorin [9], [M+H]^+^ m/z 193 for ferulic acid [10], [M+Na]^+^ m/z 441 for liquiritin [11], [M-H]^−^ m/z 579.1 for naringin [12, 13], [M-H]^−^ m/z 609.4 for hesperidin [14], [M-H]^−^ m/z 255 for isoliquiritigenin [11], [M-H]^−^ m/z 611.0 for Neohesperidinn [14], [M+H]^+^ m/z 218 for *α*-cyperone [15], [M-H]^−^ m/z 469.5 for 18*β*-glycyrrhizic acid [16], and [M+H]^+^ m/z 278.0 for the unknown targeted peak. The retention time, UV spectra, and the target ions from the chromatographic peaks obtained from blank plasma, ABCs-contained plasma, CSS, various herb, and a special fraction collected from Fructus Aurantii (FA) by a preparative chromatographic system are provided in Section 2.4 and were compared with the known reference chemicals. The unknown compound and its parent herb were validated via excluding peaks that corresponded to known compounds and the parent herb, which was FA.

### 2.4. Isolation and Identification of the Unknown Compound

#### 2.4.1. FA Sample Preparation and Extraction

The air-dried and powered fructification of FA samples (2000 g) were first soaked in 16000 mL of 95% ethanol for 8 h and then extracted 3 times with the above solvent under reflux in a 90°C water bath for 1.5 h. After removing the organic solvent from the blended solution in vacuo, an ethanol extract (500 g, wet weight) was performed. The latter was reextracted once with 6000 mL of ligarine to remove hyperpolar substances. The ligarine extract concentrated in vacuo was extracted 3 times with acetoacetate under reflux heating for 2 h. After concentrating the acetoacetate extract in rotary evaporation, 50 g of residue dissolved in 50 mL acetone was subjected to a silica gel CC (15 × 5 cm i.d.) and eluted with a gradient of ligarine/C_4_H_8_O_2_ (100 : 0 → 50 : 50) to afford one fraction. The eluted extract was concentrated to 4420 mg sample in vacuo. The 4420 mg sample was dissolved in 600 mL of 50% methanol to yield the crude extract.

#### 2.4.2. Isolation and Purification of an Unknown Compound

A waters Deltaprep Preparative Chromatography system (Milford, MA) with Symmetryprep C_18_ (7 *μ*m, 19 mm × 150 mm) column, a 7752i hand sampler, a PDA optical detector for ultraviolet wavelengths (190~500 nm), and a fraction collector was used along with the following separating conditions: linear gradient CH_3_OH(A)/H_2_O (B) (20 : 80 → 50 : 50): 0~10 min, 20% A; 10~20 min, 20~50% A; 20~44 min, 50% A; flow rate: 10 mL/min; column temperature: 25°C.

The supernatant of the FA extract (5 mL) obtained in Section 2.4.1 following centrifugation was filtered through 0.22 *μ*m film and subsequently isolated and purified by preparative HPLC to obtain a special eluting fraction with a specific retention time selected. The fraction was dried under rotary evaporation at 50°C.

### 2.5. Identification of the Unknown Compound

**Figure 1:**
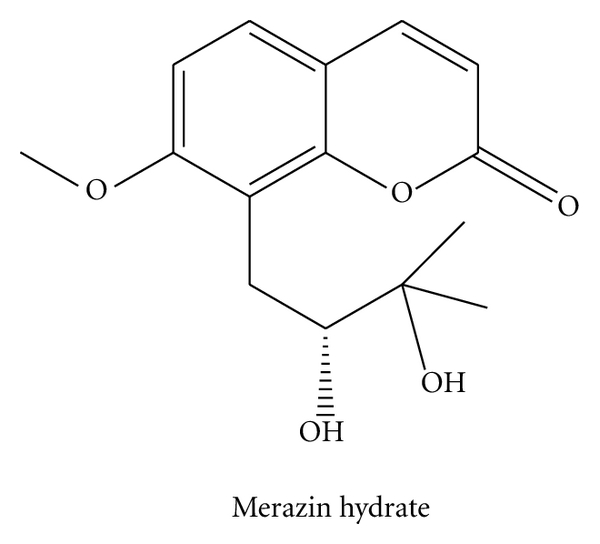
The chemical structure of Merazin hydrate.

The spectral data provided in Section 3.2 indicated that this compound was Meranzin hydrate (Figure 1), which has been isolated for the first time from FA.

### 2.6. Antidepressive Comparison of MH and FA or CSS

Forty-eight male Sprague-Dawley rats (200–240 g) from SLAC (Shanghai, China) conforming to the Regulations for the Administration of Affairs Concerning Experimental Animals (1988) were approved by the Animal Experimental Center for Central South University (Changsha, China). The rats were housed in a temperature-controlled facility, with a 12 h light/dark cycle, and had unlimited access to food and water for 7 days.

Rats were randomly assigned to groups as follows (*n* = 6): (1) vehicle: rats were gavaged 0.9% NaCl at one time; (2) MH: rats were acutely gavaged a water solution containing MH (7 and 14 mg/kg) whose low dose induced a slight effect and the high dose had a more dramatic effect; (3) CSS: rats were acutely dosed with 10 and 30 g/kg of CSS extract consistent with a previously reported dose [17]; (4) FA: rats were rapidly gavaged an aqueous solution of FA (10 and 20 g/kg) [10, 18]; (5) positive control: Fluoxetine (20 mg/kg) with a solution concentration of 0.66 mg/mL was orally administered to the rats [19]. The dosed volumes of vehicle, MH, CSS, FA and Fluoxetine were 1.5 mL/100 g for the rats [10]. The solvents here and in Section 2.3. were prepared using distilled water.

A forced swimming test (FST) was performed according to a previously published protocol [20]. The rats were individually forced to swim twice at 24 h intervals in a cylinder (40 cm high, 18 cm in diameter) filled with water (25°C) up to 15 cm deep. A 15 min preswimming period was followed 24 h later by a 5 min test period during which scoring was performed. The behavioral responses were scored every 5 s based on swimming behavioral criteria; each rat was judged to be immobile when it ceased struggling and remained floating motionless in the water, making only those movements necessary to keep its head above water.

In the Open-field test (OFT), each rat was individually placed into the center of an open field apparatus (40 cm height, 77 cm × 77 cm base divided into 49 squares 11 cm × 11 cm each) to measure the locomotor activity (LMA) for 5 min. The test was performed between 8 : 00 and 12 : 00. A 60 W light bulb provided the only source of illumination in the testing room. The crossings number (CN, i.e., a rat stepping from one square to another with its rear legs) was regarded as the measurement parameter.

### 2.7. Statistical Analysis

All data were expressed as mean ± standard deviation. A database was set up with SPSS 15.0 software package (SPSS Inc. Chicago, USA). The differences between two groups were analyzed by one-way ANOVA. A probability of less than 0.05 was considered to be statistically significant.

## 3. Results

### 3.1. Determination of an Unknown Peak in Plasma, CSS, and FA

**Figure 2:**
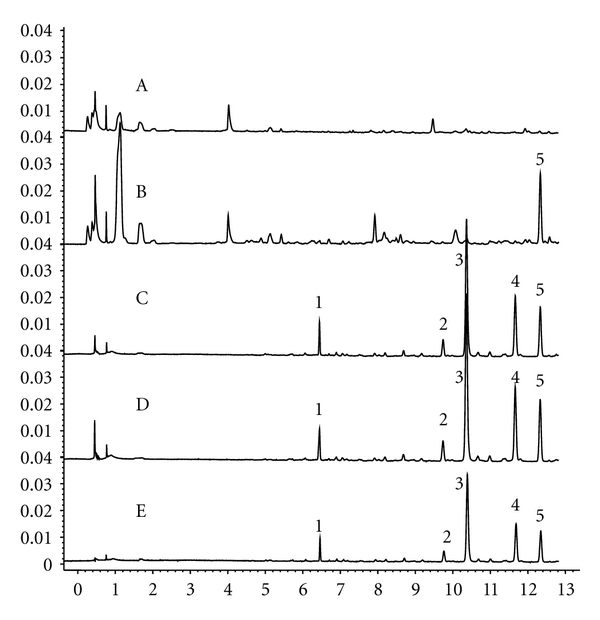
Typical chromatograms MH. (A) chromatogram of a blank plasma sample; (B) chromatogram of a plasma sample of patient with depression taken 30 min after oral administration of CSS; (C) chromatogram of CSS; (D) chromatogram of FA; (E) chromatogram of (1) Hesperidin, (2) Naringin, (3) Neohesperidin, (4) Narirutin, and (5) Merazin hydrate.

In Figure 2, peak 5 was well separated using 280 nm UV light. Peak 5 was absent in blank plasma (A) while present in the samples of plasma (B) which contained absorbed compounds derived from CSS. CSS and FA also contained this peak as shown in Figure 2 (C and D). Here, retention times (12.19~12.21 min) and UV wavelength of peak 5 were extremely similar (Figure 2). All of the target ions from peak 5 were the same ([M+H]^+^ m/z 278.0). Because there was no corresponding reference compound for peak 5, peak 5 must correspond to the unknown compound from FA.

**Figure 3:**
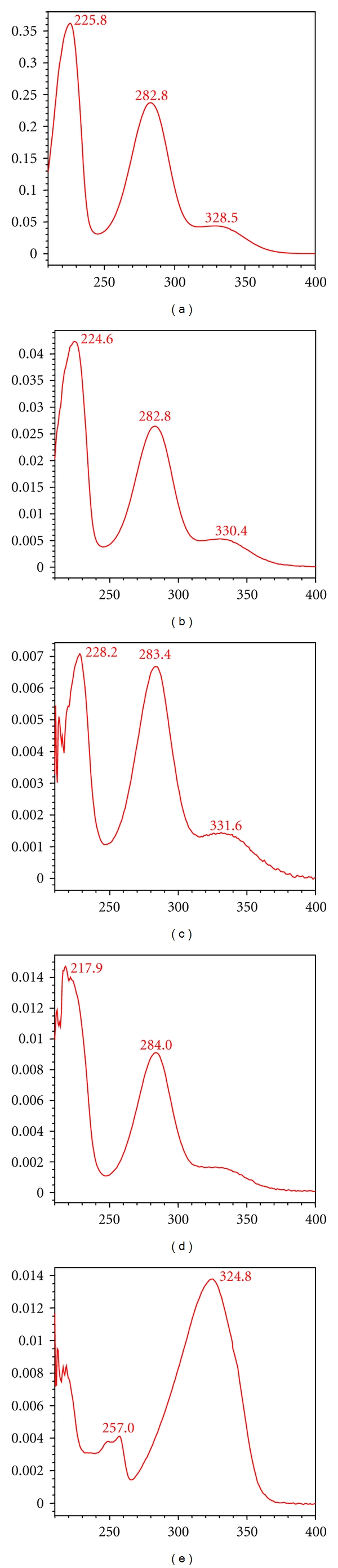
UV-absorptive spectrum of the five compounds: (a) Hesperidin, (b) Naringin, (c) Neohesperidin, (d) Narirutin, and (e) Merazin hydrate.

 The peaks of 1–4 from FA (Figure 2 D) were similar to reference compounds with regard to retention time and UV wavelength (Figure 2 E). They were hesperidin, narirutin, naringin, and Neohesperidinn, respectively. The retention time, UV profile (Figure 3), and target ion of the other peaks from CSS all corresponded to the reference compounds described in Section 2.1 (data not shown).

### 3.2. Extraction and Identification of Unknown Compound

**Figure 4:**
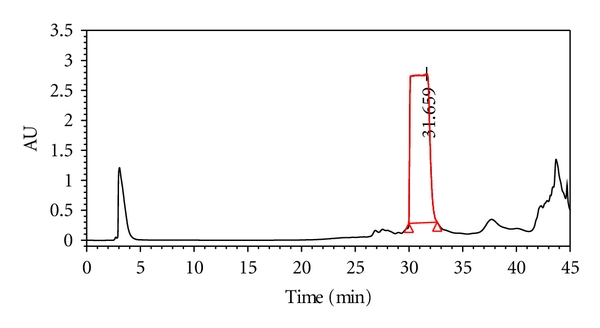
The preparative chromatogram of FA. 31.659 min peak was eluted and purified.

As shown in Figure 4, the peak (31.659 min) of FA was eluted using preparative chromatography, concentrated, and purified to obtain 190.63 mg of pure entity (the purity > 98%). The white crystalline material was identified as meranzin hydrate as shown in Figure 1.

 Results from UV, IR, MS, and NMR analysis are as follows:

UV max (EtOH) nm: 212, 226, 262, 306 nm; IR (KBr) cm-1: 3200 (OH), 1710 (C=O), 1600, 1500(Ar), 1435(CH), 1250(C-O); 1H NMR (1H-1H COSY) *δ*ppm: 7.95 (1H, d, *J *= 9.5 Hz, 4-H), 7.53 (1H, d, *J* = 7.5 Hz, 5-H), 7.03 (1H, d, *J* = 7.5 Hz, 6-H), 6.24 (1H, d, *J* = 9.5 Hz, 3-H), 3.87 (3H, s, OCH_3_), 4.15 (1H, brs, OH), 4.00 (1H, d, *J* = 5.9 Hz, OH), 3.51 (1H, dd, *J* = 10.0, 2.5 Hz, 2′-H), 2.90 (1H, dd, *J* = 12.0, 10.0 Hz, 1′-Ha), 2.80 (1H, dd, *J* = 12.0, 2.5 Hz, 1′-Hb), 1.14 (6H, s, 2 × CH_3_); 13C NMR (100.6 MHz, CDCl_3_) *δ*: 26 (C5′), 25.5 (C4′), 72.5 (C3′), 76.8 (C2′), 25.5 (C1′), 56.5 (O-CH_3_), 117.4 (C10), 153.6 (C9), 108.3 (C8), 161 (C7), 113 (C6), 127.4 (C5), 145.2 (C4), 112.5 (C3), 161.0 (C2). EIMS m/z (%): 278 (M+), 263, 245, 220, 189, 177(100), 131, 109. (Found: C, 64.78; H, 6. 48. Calc. for C_15_H_18_O_5_: C, 64.75; H, 6.53). These data are consistent with a previously published work [21].

### 3.3. Antidepressive Comparison between MH and FA or CSS

The immobility time (s) and the number of crossings (n) obtained for the rats that were forced to swim were compared with sham (not swimming). The immobility time was found to increase significantly (immobility time, 120.9 ± 21.2 versus 168.2 ± 24.6), while the number of crossings was reduced (122.4 ± 19.1 versus 94.2 ± 18.7) for the drug treatment groups in these studies. All of these data indicated the success of depressive model.

**Figure 5:**
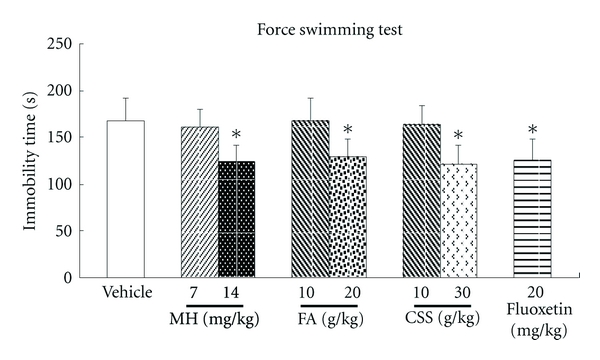
Effect of treatment with MH, FA, and CSS on the immobility of rats in the FST. Rats were treated with 0.9% saline (10 mL/kg) (Vehicle), MH (7, 14 mg/kg), FA (10, 20 g/kg), CSS (10, 30 g/kg), and Fluoxetine (20 mg/kg). *P < 0.05 compared with the vehicle group.

Compared with the vehicle group, subacute administration of MH, FA, and CSS at a high dosage amount, dramatically reduced the immobility time (s) (168.2 ± 24.6 versus 123.3 ± 19.6, 128.6 ± 20.6, or 120.7 ± 21.4; all *P* values were <0.05) as shown in Figure 5, which is similar to Fluoxetine (125.4 ± 23.6).

**Figure 6:**
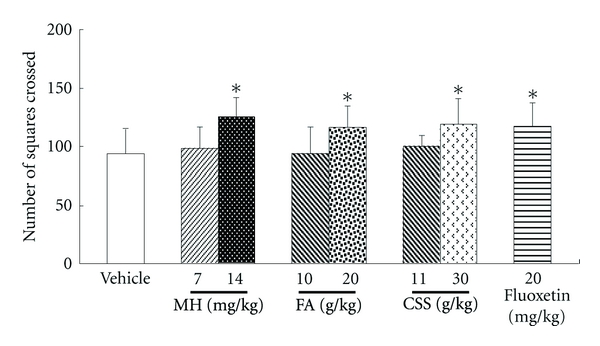
Effect of treatment with MH, FA, and CSS on locomotor activity evaluated in the open field test. Rats were treated with 0.9% saline (10 mL/kg) (control), MH (7, 14 mg/kg), FA (10, 20 g/kg), CSS (11, 30 g/kg) and Fluoxetine (20 mg/kg). *P < 0.05 compared with the vehicle group.

 When gavaged with a high dose of MH, FA, and CSS, all of the rats increased the number of crossings (125 ± 17,  116 ± 19  and 119 ± 22, resp.) compared with the vehicle sample (94 ± 21; all *P* values were <0.05) as shown in Figure 6. These data were similar to Fluoxetine (117 ± 21).

## 4. Discussion

MH, a compound isolated from FA for the first time by us (Chinese patent was applied in 2008 and authorized in 2011), and some of its further researches were also performed and published [22, 23]. MH was readily absorbable into the blood stream and could potentially lead to antidepressive effects which are similar to FA or CSS. An unknown chromatographic peak in patient blood following oral CSS was targeted to ascertain its herbal source (Figure 2). Following isolation, the structure was identified using spectral data and its antidepressive effects were compared with FA or CSS (Figures 4 and 5). These steps illustrate the EAGI method. This approach and its application can help to avoid the high rate of failure for drug R&D due to poor pharmacokinetics [3] and efficacy [1, 24].

In chromatogram of Figure 2, MH was isolated from FA via targeting absorption peak 5. Its absorption was unique and its structure was the only unknown peak. Retention times, UV spectra, and target ions of peak 5 in plasma, CSS, and FA were nearly identical (Figures 2 and 3) and did not corresponded to known reference chemicals. Using UV, IR, MS and NMR, the structure of this compound was elucidated (Figure 1), which was consistent with previously published work [21]. MH has been isolated from FA for the first time. Previously, MH was found in the peel of Phellolophium madagascariense Baker [25], Magydaris tomentosa [26], Citrus maxima fruit [27], Murraya paniculata [28], and Triphasia trifolia [21]. Is it surprising that MH has not been previously isolated from FA [29, 30]. From Figure 2, the four known compounds exhibited poor absorption. Tedious isolations of the known compounds and a potentially high failure rate for drug R&D had previously occurred using the AGI method [2, 3].

EAGI was used to make in vivo antidepressive comparisons between MH and its parent FA or CSS (Figures 4 and 5) based on clinical results. For several centuries, CSS has been used to clinically attenuate mood disorders similar to depression or the TCM syndrome known as “liver depression” [6]. Such comparisons resulted in the production of antidepressive MH in the present study. When gavaged acutely at high doses in rats, MH, FA, and CSS all significantly reduced immobility time by 23.5–28.2% (*P* < 0.05) and increased the numbers of crossing by 23.4–33.0% (*P* < 0.05), which is consistent with the results obtained for Fluoxetine. The traditional clinical use of CSS played an important role in discovering the novel antidepressive, MH. Previous research had only implicated its use in anticancer [25], antibacterial, and anticoagulation [26] treatments. In contrast, AGI mainly involves the analysis in vitro activity with less focus on holistic efficacy, which is consistent with the high failure rate of drug R&D due to poor efficacy [1, 24].

In conclusion, MH, a compound isolated from FA for the first time via BAGI, was detected by its structure spectral data, and its antidepressive effects were found to be similar to FA and CSS. This result highlights the hit-to-lead optimization of a drug candidate [31] and helped to elucidate the antidepressive mechanism of CSS.
